# Contraceptive implant continuation among postpartum women at tertiary care center

**DOI:** 10.12669/pjms.42.1.12408

**Published:** 2026-01

**Authors:** Tayyiba Wasim, Natasha Bushra, Tahira Nasreen, Afshan Shahid

**Affiliations:** 1 Tayyiba Wasim, FCPS, FRCOG Department of Obstetrics and Gynaecology, SIMS, Services Hospital, Lahore, Pakistan; 2 Natasha Bushra, FCPS Department of Obstetrics and Gynecology, Post Graduate Medical Institute, Ameer-ud-Din Medical College, Lahore, Pakistan; 3 Tahira Nasreen, FCPS Department of Obstetrics and Gynaecology, SIMS, Services Hospital, Lahore, Pakistan; 4 Afshan Shahid, FCPS Department of Community Medicine, SIMS, Services Hospital, Lahore, Pakistan

**Keywords:** Continuation rate, Etonorgestrel implant, Postpartum contraception

## Abstract

**Objective:**

To evaluate the continuation rates and factors influencing the discontinuation of postpartum etonorgestrel contraceptive implants over a three-year period.

**Methodology:**

A prospective cohort study was conducted from November 2017 to December 2022 in the Department of Obstetrics and Gynecology, Services Hospital, Lahore, Pakistan. The patients coming to OPD and emergency were counselled for insertion of long-acting reversible contraception. Those who opted for postpartum etonorgestrel implants were enrolled after informed consent and implant was placed within 48 hours of birth. The patients were followed for three years post insertion. The primary outcome was the continuation rate at three years, and secondary outcomes included reasons for discontinuation. Statistical analysis was performed using SPSS version 23, and p-values < 0.05 were considered statistically significant.

**Results:**

Of 424 participants the cumulative continuation rates for year 1,2 and 3 were 91.9%, 82.7% and 75% respectively and 106 (25%) removals at the end of year 3. Doctors counselling played role in choosing implant in 177(41.7%) patients. Continuation was higher among women with prior contraceptive knowledge AOR: 3.73; 95% CI (1.20-6.21), previous contraceptive use AOR: 8.1; 95%CI (4.2-31.1), and antenatal counselling AOR: 2.24; 95% CI (1.16-3.54). The most common reasons for discontinuation were side effects in 77(45.2%) and family pressure 55(32.3%) patients. Women with no side effects were 18 times more likely to continue AOR: 18.1; 95% CI (7.5-48).

**Conclusion:**

The postpartum insertion of etonorgestrel implant has high continuation rates. Side effects of bleeding irregularities and family pressure were main reasons for discontinuation.

## INTRODUCTION

Short interpregnancy intervals pose significant risks to both maternal and child health, leading to increased maternal and perinatal mortality. The World Health Organization (WHO) and the American College of Obstetricians and Gynecologists (ACOG) recommend an optimal pregnancy interval of at least 24 months to reduce these risks.^1^,^2^ Pakistan, the fifth most populous country in the world with an estimated population of over 240 million faces considerable challenges due to its rapid population growth.^3^ The population explosion exerts pressure on resources such as healthcare, education, food, and water supply. Insufficient family planning and limited access to birth control are major contributors to this issue.

Since 2007, Pakistan’s Contraceptive Prevalence Rate (CPR) has remained stagnant within the 30-35% range, highlighting a significant unmet need for contraception.^4^ Postpartum contraception defined as immediate contraception after childbirth, has been identified as an effective strategy to address this need. Among the most effective contraceptive methods are Long-Acting Reversible Contraceptives (LARC), including intrauterine devices (IUDs) and contraceptive implants.^5^ The etonogestrel implant, a subdermal hormonal rod, provides highly effective pregnancy prevention for up to three years, with an annual pregnancy rate of only 0.05%.^6^

Measuring 4 cm in length and 2 mm in width, it is inserted just beneath the skin of the upper arm by a trained healthcare provider and requires a minor surgical incision for removal. The implant’s active ingredient, etonogestrel, is a synthetic progestin that primarily prevents ovulation by suppressing the release of luteinizing hormone. One-year continuation rates range from 57% to 97%, with 44% to 95% of users continuing the method into the second year, and 25% to 78% by the third year.^7^ Given the importance of family planning in Pakistan and the potential for influencing patient choices, this study aimed to investigate the continuation rates of the etonogestrel implant when inserted postpartum.

Additionally, it sought to identify the factors contributing to early discontinuation within a specific clinical context. To the best of our knowledge, this is the first study to report on the postpartum insertion and continuation of the contraceptive implant in Pakistan. The results of this study could provide valuable insights for healthcare providers and patients, potentially leading to increased uptake of modern contraceptive methods in the country.

## METHODOLOGY

This prospective cohort study was conducted in the Department of Obstetrics and Gynecology, Services Hospital, Lahore, Pakistan, from November 1, 2017, to December 31, 2022.

### Ethical Approval:

It was obtained from the Institutional Review Board (Ref No. IRB/2017/373/SIMS, dated: October 28, 2017). The contraceptive supplies were provided free of cost by Government of Punjab.

All pregnant patients presenting for antenatal care were counseled regarding the need for contraception using the GATHER technique. Women willing to use long-acting reversible contraception (LARC) with subdermal implant (Implanon) were informed about the potential benefits and risks. After obtaining informed consent, they were enrolled in the study. Antenatal cards of consenting participants were stamped to indicate their willingness. Women who opted for implants received insertions within 48 hours of delivery by trained experts. Details such as the date of insertion and the timeline for removal were documented. Participants were counseled regarding possible side effects, including menstrual irregularities, such as irregular or heavy bleeding, as well as other symptoms like headache, weight gain, acne, and breast pain.

Participants were advised to attend follow-up visits at six months and subsequently at 1-, 2-, and three years’ post-insertion. Demographic data and any side effects were recorded. Participants who missed scheduled follow-ups were contacted via telephone. Those who failed to follow up despite reminders were excluded from the study. The primary outcome variable was the continuation rate of the etonogestrel implant at three years, defined as the proportion of women who initiated and continued using the contraceptive method over the study period. Secondary outcomes included reasons for discontinuation. Data were analyzed using SPSS version 23. Qualitative variables were expressed as proportions with 95% confidence intervals. Comparisons between qualitative variables were performed using the chi-square test or Fisher’s exact test, as appropriate. A p-value ≤0.05 was considered statistically significant.

## RESULTS

**Fig.1:**
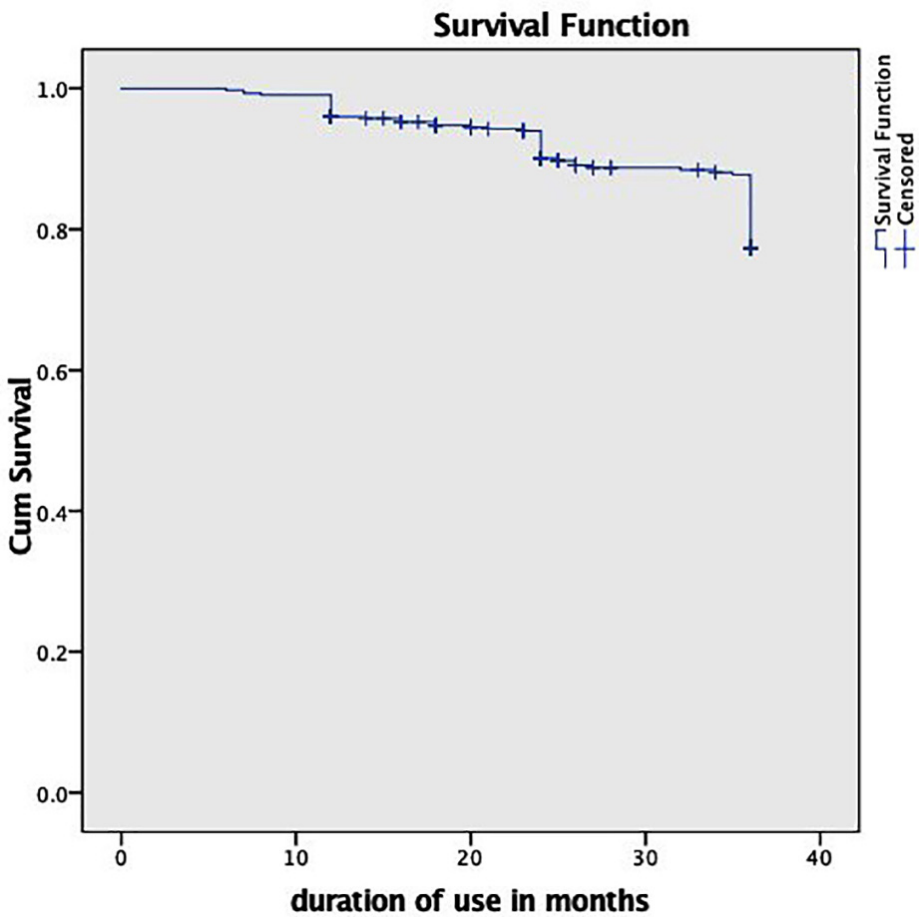
Kaplan Meier survival estimate curve for implanon continuation and discontinuation

In this study, a total of 424 women were followed up for three years. Out of 424 participants the cumulative continuation rates for year one, two and three were 390 (91.9%), 351 (82.7%) and 318 (75%) respectively (Fig.1). The total Implant removals at the end of year 3 were 106(25%) with no Implanon removed during the first six months of insertion. The majority (53.3%) were 21-30 years of age and 202 (47.6%) had no formal education. A total of 225 (53.1%) participants were aware of contraceptive methods. Among the participants, 183 (43.2%) reported using contraception previously. Reasons for not using contraception included family pressure (n=139, 57.6%), religious concerns (n=110, 45.5%), myths and misconceptions (n=189, 78.4%), fear of infertility (n=98, 40.7%), and being primigravida (n=81, 33.6%) (Table-I).

**Table-I:** Socio demographic characteristics (n=424).

Variables	N (%age)
***Age (years)***	
≤ 20	77 (18.2)
21-30	226(53.3)
31-40	121(28.5)
***Education***	
Illiterate	202 (47.6%)
Primary	85 (20.0%)
Secondary	92(21.7%)
Graduates	45 (10.6%)
***Family Income***	
≤ 50,000 Rs	260(61.3)
> 50,000 Rs	164(38.7)
***Parity***	
≤ 3	151(35.6)
>3	273(64.4)
***Knowledge about contraceptives***	
Yes	345(81.1)
No	80(18.8)
***Contraceptive used previously***	
Yes	183(43.2)
No	241(56.8)
***Reason for not using any contraception (n=241)***	
Family pressure	139(57.6)
Religious issues	110(45.5)
Myths	189(78.4)
Fear of infertility	98(40.6)
Primi Gravida	81(33.6)

Around 177 (41.7%) chose Implant after attending counseling sessions where doctors explained its benefits. Family support influenced 88 (20.8%) participants to develop favorable attitudes toward its use. The main reasons for discontinuation with Implant were side effects in 77 (45.2%) and family pressure in 55 (32.3%) patients. The most common side effects included bleeding abnormalities amenorrhea/PV spotting 93(25.6%), heavy menstrual bleeding (83;22.9%), breast tenderness (77;21.2%), weight gain (57;15.7%), and headache/mood changes (53;14.6%) (Table-II).

**Table-II:** Implant use related factors in females (n=424).

Variables	N (%age)
***Duration of Implant use (years)***	
Duration of use at year 1	390(92%)
Duration of use at year 2	352(83%)
Duration of use at year 3	318(75%)
Minimum duration	6 months
Maximum duration	3 years
***Reason for choosing Implant***	
Long acting	121(28.5)
Family choice	88(20.8)
Doctor counseling	177(41.7)
Single visit convenience	38(9.0)
***Reason for Discontinuation of Implant n= 170***	
Side effects	77(45.2)
Family pressure	55(32.3)
Desire to conceive	25(14.7)
Religion	6(3.5)
***Types of side effects with Implant***	
Heavy menstrual bleeding	83(22.9%)
Amenorhea/PV spotting Weight gain	93(25.6%) 57(15.7%)
Headache/Mood changes	53(14.6%)
Breast tenderness	77(21.2%)

Factors affecting the utilization of Implant were analyzed using bivariate and logistic regression analyses. On bivariate analysis, variables such as age, knowledge about birth spacing, prior contraceptive use, complaints during Implant use, having more than two complaints, and antenatal counseling were associated with current Implant use. However, on logistic regression analysis, only knowledge about birth spacing, prior contraceptive use, and the presence of complaints with Implant use remained statistically significant. Women with knowledge about birth spacing were more than three times as likely to use Implant compared to those without such knowledge (AOR: 3.73; 95% CI: (1.20-6.21). Similarly, women who had previously used any contraceptive method were over eight times more likely to use Implant compared to those who had never used contraceptives (AOR; 8.1, 95% CI: (4.2-31.1). Women with no side effects, were eighteen times more likely to use Implant (AOR; 18.1, 95% CI: (7.5-48) (Table-III).

**Table-III:** Factors that affect utilization of Implant (n=424).

Characteristics	Current use of implant		Odd’s Ratio	
	Using	Not using	COR(CI)	AOR(CI)
***Age***				
< 30 years	229(67.3)	74(32.7)	1	
>30 years	118(97.5)	3(2.5)	3.4(2.1-5.2)*	1.2(.86-5.3)
***Previous Knowledge about birth spacing***				
No	166(73.8)	59(26.2)	1	3.73(1.20-6.21)*
Yes	181(91.0)	18(9.07)	3.57(2.02-6.30)*	
***Any contraception used previously***				
No	125(68.3)	58(31.7)	1	
Yes	222(92.1)	19(7.9)	4.24(3.6-28.8)*	8.1(4.2-31.1)*
***Side effects with implant use***				
No	212(87.6)	30(12.4)	1	
Yes	136(74.4)	47(25.8)	2.4(1.4-4.08)*	18.1(7.5-48)*
***Number of side effects***				
>2	296(83.8)	58(16.7)	1	
<2	2(27.3)	7(72.7)	6.2(3.4-7.8)*	1.02(.187-5.5)
***Counseling***				
Emergency counseling	224(90.0)	25(10.0)	1	
Antenatal counseling	123(70.3)	52(29.7)	3.78(2.2-6.4)*	2.24(.116-3.54)

* statistically significant p <.05, adjusted for education, family income and parity.

## DISCUSSION

The findings from our study provide significant insights into the factors influencing the utilization and continuation of the postpartum etonogestrel implant. Our study shows that 81.1% women had knowledge of contraceptive methods but only 43.3% used any method of contraception. Reasons identified in our study were myths related to contraception, family pressure and religious concerns. Similar reasons contributing to the hesitation in contraceptive use are reported from other studies from Pakistan.^8^-^10^ Work for raising CPR needs to be done at war footing addressing contraceptive availability, limited awareness, familial, social barriers, and clinical concerns related to contraception. Bangladesh has a sevenfold increase in its (CPR) in less than forty years from 8% in 1975 to 62% in 2014working in right direction.^11^ Postpartum insertion of LARC offers several advantages, including the convenience of no follow-up visits, reduced user dependency, and safety during breast feeding. The postpartum period is a window of opportunity to initiate contraception.

The study shows continuation rate of 59.9% over three years which is promising in context of our CPR. Similar continuation rates are reported from other studies as well. A study reporting postpartum insertion of IUCD and implant reported 96% continuation in one year.^12^ Another study shows continuation of 70% at three years^13^ while study from Netherland report continuation of 25 months.^14^ A study in India by reported a continuation rate of 60% of LARC at one year.^15^ Our study revealed that knowledge about birth spacing and prior contraceptive use significantly influenced the likelihood of using the etonogestrel implant. A Study from Gilgit emphasized the distance from health center affecting continuation^16^ while younger age affected continuation in study from Brazil.^17^

The role of antenatal counseling emerged as a significant factor in our study, with women who received such counseling showing higher continuation rates. This finding aligns with a systematic review by Bansal et al and Dev et al which concluded that effective counseling during antenatal visits significantly enhances the likelihood of adopting and continuing contraceptive methods postpartum.^18^,^19^ The importance of integrating family planning education into antenatal care cannot be overstated, as it addresses the unmet needs for family planning and empowers women to make informed choices.

The presence of side effects played a crucial role in discontinuation of the implant in our study. This is similar to the evidence of almost all studies reported.^14^,^20^,^21^ Our results highlight the need for healthcare providers to offer comprehensive counseling about potential side effects and management strategies to improve user satisfaction and continuation rates. Family pressure was another reason for discontinuation. In Pakistan, husband and mother-in-law have a crucial role in deciding contraceptive choices as is reported by other studies as well.^9^,^22^ There is dire need to sensitize the public about the importance of birth spacing and its link to mother and child health. Husband is main deciding force for use of contraception as reported in studies from other Low middle-income countries (LMIC).^23^ That emphasize the need of male mobilizers to be included in counselling.

In Pakistan, contraceptive decisions are shaped by socio-cultural hierarchy, with husbands and elders often influencing women’s choices. Myths about infertility, menstrual irregularities, and religious permissibility remain common, reinforced by limited counselling, poor access to accurate information, and minimal male involvement. Systemic barriers including staff shortages, overburdened facilities, and inconsistent contraceptive supply further contribute to discontinuation.^24^ Moreover, healthcare system constraints such as overburdened facilities, shortage of trained counsellors, and inconsistent availability of contraceptive commodities further contribute to discontinuation rates.^25^

Culturally sensitive strategies are needed to address these barriers. Couple-based education, involvement of male health workers, and engagement of religious leaders can help dispel misconceptions. Integrating structured contraceptive counselling within routine antenatal and postnatal care by trained female providers can build trust, improve continuation, and promote reproductive health equity in Pakistan.^26^

This study provides the first three-year prospective data on postpartum etonogestrel implant continuation in Pakistan. Its strength lies in the longitudinal design and systematic follow-up, offering context-specific insights into counselling, prior contraceptive use, and socio-cultural factors. The findings have clinical relevance for strengthening antenatal and couple-based counselling and improving side-effect management. Multi-center and qualitative studies are recommended to guide future policy and program development.

### Limitations:

This was the first prospective cohort study from Pakistan evaluating postpartum etonogestrel implant continuation with three-year follow-up. Being a single-center study with a modest sample size, the findings may not be generalizable to all healthcare settings. Recall bias cannot be ruled out since information on side effects was self-reported. Moreover, some loss to follow-up occurred, which may have affected the accuracy of continuation rates. Data regarding male partner perspectives and socioeconomic variations were not included, which limits understanding of household-level decision dynamics influencing contraceptive use.

## CONCLUSION

Postpartum insertion of the etonogestrel implant shows high continuation at three years. Women counseled antenatally and those with prior contraceptive awareness were more likely to continue use, while side effects and family pressure led to discontinuation. Policymakers should integrate male-focused and couple counselling, strengthen antenatal family planning education, and implement side-effect management protocols to enhance continuation and satisfaction.

### Recommendations:

Future multicenter studies with larger and more diverse populations, including qualitative components, are recommended to validate and enrich these findings.

### Author’s Contribution:

**TW:** Conceived, designed, final manuscript editing and is responsible for integrity of research.

**NU:** Did initial manuscript writing. Critical Review.

**TN and AS:** Did data collection and statistical analysis.

All authors have read and approved the final version.
